# Association between family history, mutation locations, and prevalence of *BRCA1* or *2* mutations in ovarian cancer patients

**DOI:** 10.1002/cam4.2000

**Published:** 2019-03-01

**Authors:** Christian F. Singer, Yen Y. Tan, Daniela Muhr, Christine Rappaport, Daphne Gschwantler‐Kaulich, Christoph Grimm, Stephan Polterauer, Georg Pfeiler, Andreas Berger, Muy‐Kheng M. Tea

**Affiliations:** ^1^ Department of OB/GYN and Comprehensive Cancer Center Medical University of Vienna Vienna Austria

**Keywords:** BRCA, family history, mutation locations, ovarian cancer, pedigree

## Abstract

We investigated the prevalence of germline *BRCA* mutations in a population‐based cohort of Austrian women diagnosed with ovarian cancer and its association with family history of cancer. We prospectively collected family pedigrees of 443 Austrian ovarian cancer patients who had been tested for the presence of a germline *BRCA* or *2* mutations and correlated the familial breast and ovarian cancer burden with the prevalence of *BRCA* mutations and disease onset. The probability of carrying a g*BRCA* mutation in patients without family history of cancer is 14% (95% CI 9%‐22%), as opposed to 45% (95% CI 31%‐59%) of patients with at least one family member with ovarian cancer, and 47% (95% CI 40%‐54%) if other relatives have developed breast cancer. If both breast and ovarian cancer are diagnosed in the family, the probability of carrying a germline *BRCA1* or *2* mutations is 60% (95% CI 50%‐68%). germline *BRCA1* or mutations in families with ovarian cancer only are commonly located in the Ovarian Cancer Cluster Regions when compared to families with both breast and ovarian cancer (*P* = 0.001, and *P* = 0.020, respectively). While g*BRCA mutation* carriers with ovarian cancer do not have a significantly different age at onset than patients with a family history of cancer, g*BRCA1* carriers in general have an earlier onset than g*BRCA2* carriers (*P *= 0.002) and patients without a mutation (*P *= 0.006). The rate of germline *BRCA1* or *2* mutations in ovarian cancer patients without a family history or breast or ovarian cancer is low. However, in women with additional family members affected, the prevalence is considerably higher than previously reported.

## 1 INTRODUCTION

Germline mutations of *BRCA1* and *BRCA2* confer a particularly high life‐time risk of developing breast (BC) and ovarian (OC) cancer.^1^, ^2^ Following an autosomal dominant pattern of inheritance, they are passed on to subsequent generations with a probability of 50% and consequently result in familial aggregation of disease. It is therefore not surprising that families which comprise multiple cases of breast and/or ovarian cancer exhibit a higher prevalence of *BRCA1* and *BRCA2* mutations than families without affected members.^3^ Nevertheless, it has been estimated that 44% of the detected germline mutations also occur in women without a family history of cancer.^4^ Population‐based studies have suggested that *BRCA1* and *BRCA2* germline mutations are present in approximately 15% of all OC cases, but prevalence data vary significantly due to different analytical sensitivity and the sampled population.^5^, ^6^, ^7^ While traditionally *BRCA* mutation analysis has been limited to OC patients with a significant family history of cancer, the availability of PARP inhibitors and its efficacy in platin‐sensitive recurrent OC in women with *BRCA1 and BRCA2* mutations now mandates testing of women with epithelial OC.^8^, ^9^ This strategy is supported by national and international guidelines.^10^ Since descendants of OC patients now increasingly request for their *BRCA* status in order to assess their individual risk, it is important to provide prevalence data in relation to a particular family history.^11^, ^12^

We have therefore analyzed the prevalence of *BRCA1 *and *2 *germline mutations in a population‐based cohort of 443 Austrian women with OC in relation to their family history. Patients were prospectively followed up for incident familial breast and ovarian cancer cases. In this cohort, we also have evaluated the age of disease onset in mutation carriers in relation to their family history and compared it to women with no germline mutation.

## 2 MATERIALS AND METHODS

### 2.1 Subjects and Methods

In Austria, genetic testing for *BRCA* mutation has been conducted in Vienna General Hospital since 1995. Denaturing high‐performance liquid chromatography (dHPLC) and Sanger sequencing were the molecular diagnostic methods used up to 2007. From 2007 to 2015, Sanger sequencing alone was used in place of dHPLC, and multiplex ligation‐dependent probe amplification (MLPA) was performed subsequently to identify large deletions or duplications. MLPA was also conducted retrospectively on patient samples collected prior to 2007. From 2015 onward, next‐generation sequencing is performed in the General Hospital in place of MLPA/Sanger sequencing. Detected mutations are confirmed by Sanger sequencing and MLPA, respectively. Women are offered testing if their familial history fulfills at least one of the following criteria: (a) three cases of BC below age 60, (b) two cases of BC below age 50, (c) one case of BC below age 35, (d) one BC case below age 50, (e) one case of OC at any age, (f) two cases of OC at any age, and (g) male and female BC. From 2015 onward, germline testing is also offered to all women who were diagnosed with epithelial OC regardless of family history of cancer at no cost.^13^ To identify these individuals, we searched our nationwide cohort of 6691 Austrian women (as of February 2016) who had fulfilled the selection criteria above, had provided informed consent, had undergone germline *BRCA* (g*BRCA) *mutation analysis, and had provided a comprehensive family history at the time of analysis. Patients were followed longitudinally and received regular questionnaires every 2 years in order to identify incident familial breast and ovarian cancers. This study was approved by the Ethics Committee of the Medial University of Vienna in accordance with the Declaration of Helsinki.

### 2.2 Statistical analyses

The full analysis set included 443 OC patients with or without a family history of OC and/or BC and known *BRCA* status. Family history of OC and/or BC was categorized into four groups: (a) no additional OC or BC in family, (b) at least one additional OC in family, (c) no additional OC but at least one BC in family, (d) at least one additional OC and at least one BC in family. *BRCA *status was categorized into three groups, that is, (a) *BRCA1* mutation carriers, (b) *BRCA2* mutation carriers, and (c) wild‐type non‐mutation carriers. Descriptive statistics were computed for OC patients with known *BRCA* status. *BRCA1* or *2* nucleotide positions/OC cluster regions (OCCRs) were categorized as previously described in Rebbeck et al^14^ where the putative *BRCA1* OCCR was identified from c.1380 to c.4062 (approximately exon 11) and the putative BRCA2 OCCRs from c.3249 to c.5681 including c.5946delT (c.6174delT) and c.6645‐c.7471. Ages at diagnosis and OCCRs were compared among patient groups based on their family history of cancer and their mutation type (Tables 1 and 2) using analysis of variance (ANOVA) or student's *t *test. Categorical data were compared across groups using Chi‐square or Fisher's exact test (in case of small numbers). *P‐*values less than 0.05 were considered statistically significant. Samples classified as unknown for any variables were excluded. Analyses were conducted in SPSS version 21.0 (Chicago, IL, USA).

**Table 1:** Patients characteristics by mutation type

	Total		Age at diagnosis^a^		Family history of cancer^b^							
					No BC/OC		≥1 OC, no BC		≥1 BC, no OC		≥1 BC and ≥1 OC	
	N	%	Median year	Range	N	%	N	%	N	%	N	%
*BRCA1*	143	32.3	49	32‐75	7	6.3	17	36.2	60	34.3	59	54.1
*BRCA2*	41	9.3	54	28‐74	9	8.0	4	8.5	22	12.6	6	5.5
WT/UV	64	14.4	53	18‐81	96	85.7	26	55.3	93	53.1	44	40.4
Total^c^	443				112	25.3	47	10.6	175	39.5	109	24.6

a Missing age at cancer diagnosis for 6 *BRCA1* and 2 *BRCA2* mutation carriers.

b Column percentage.

c Row percentage.

**Table 2:** Patients characteristics by mutation location

	Total		Family history of cancer^a^							
			No BC/OC		≥1 OC, no BC		≥1 BC, no OC		≥1 BC and ≥1 OC	
	N	%	N	%	N	%	N	%	N	%
*BRCA1*										
Non‐OCCR	86	60.1	3	42.9	4	23.5	40	66.7	39	66.1
OCCR	57	39.9	4	57.1	13	76.5	20	33.3	20	33.9
*BRCA2*										
Non‐OCCR	24	58.5	3	33.3	1	25.0	15	68.2	5	83.3
OCCR	17	41.5	6	66.7	3	75.0	7	31.8	1	16.7

a Column percentage.

## 3 RESULTS

### 3.1 Prevalence of germline *BRCA* or *2* mutations in OC patients

Of 443 women identified with a personal history of OC and a known *BRCA* status, g*BRCA1* mutation had been detected in 143 cases (32%; 95% Confidence Interval (CI) 28%‐37%) and g*BRCA2* mutation in 41 cases (9%; 95% CI 7%‐12%). In total, 184 (42%; 95% CI 37%‐46%) women diagnosed with OC had been found to carry a mutation in either of the two genes. The overall median follow‐up time of the cohort is 8 years (95% CI 7.1‐8.9 years).

**Figure 1:**
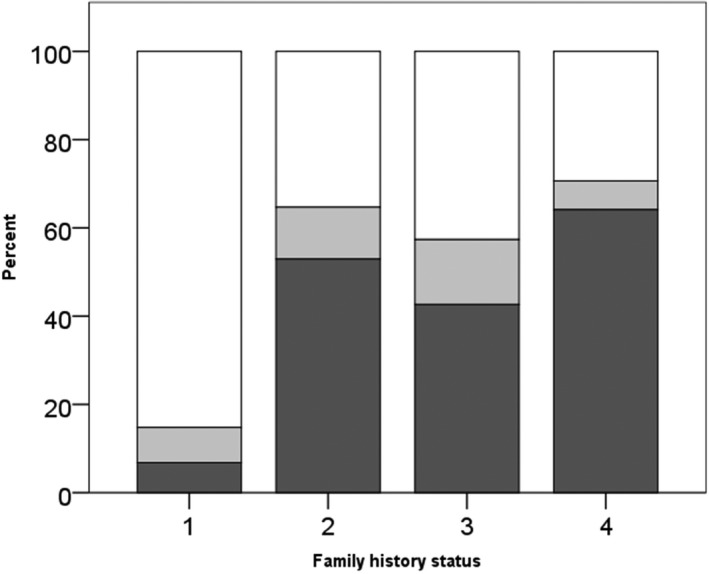
Prevalence of germline *BRCA1 *(dark gray) and *2 *(light gray) mutations and wild‐type *BRCA* (WT, white) in OC patients with: (1) no additional OC or BC in family; (2) at least one additional OC in family; (3) no additional OC but at least one BC in family; (4) at least one additional OC and at least one BC in family

We then looked at the prevalence of germline *BRCA1* or *2* mutations in ovarian cancer patients without any family member affected by OC or BC. Of 112 patients, seven patients carried a g*BRCA1* while another nine patients carried a g*BRCA2* mutation, which amounts to an overall g*BRCA* prevalence of 14% (95% CI 9%‐22%) (Figure 1). Of the 47 OC patients who had at least one other female relative diagnosed with OC but who had no BC family history, 17 carried a g*BRCA1* mutation and four others carried a g*BRCA*2 mutation. Overall, 21 of 47 patients (45%; 95% CI 31%‐59%) carried a mutation in either of the two genes. Of the 175 OC patients who had reported at least one family member with a history of BC, 60 were g*BRCA1* mutation carriers and 22 were g*BRCA2* mutation carriers, which result in an overall mutation probability of 47% (95% CI 40%‐54%). One hundred and nine OC patients were identified to have at least another relative diagnosed with OC and another with BC in the family. These women had a 54% (95% CI 45%‐63%) chance (59/109) to be a g*BRCA1* carrier, and a 6% (95% CI 3%‐11%) chance (6/109) to be a g*BRCA2* carrier, which accumulates to an overall g*BRCA* mutation probability of 60% (95% CI 50%‐68%).

### 3.2 Mutational spectrum and family history

**Figure 2:**
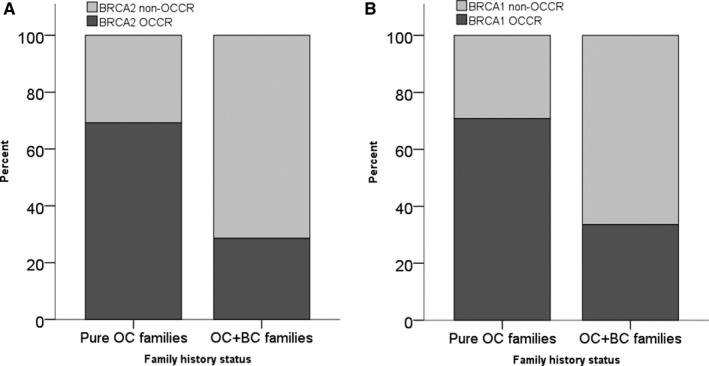
(A) Prevalence of *BRCA1* mutation in relation to their respective Ovarian Cancer Cluster Region (OCCR). (B) Prevalence of *BRCA2* mutation in relation to their respective OCCRs

We then looked at the position of germline *BRCA1* or *2* mutations in relation to their respective Ovarian Cancer Cluster Regions (OCCR). In OC patients with a g*BRCA1* mutation with or without additional OC cases in the family, who did not have a family history of BC (from here onward referred to as “pure OC families”), the majority of mutations (17/24; 71%, 95% CI 51%‐85%) were positioned in a *BRCA1* OCCR, while the remaining 29% (95% CI 15%‐49%) were located in non‐*BRCA1* OCCR regions. In OC patients with at least one BC diagnosed in the family (ie, “OC+BC families”), significantly fewer mutations (40/119; 34%, 95% CI 26%‐42%) were located in the *BRCA1* OCCR (*P* = 0.001; Chi Square test). Sixty‐six percent of mutations was found in non‐*BRCA1* OCCRs (Figure 2A), and this was independent of whether there were additional OC cases in family members or not (data not shown).

In OC patients with g*BRCA2* mutation and "pure OC families”, 69% (9/13; 95% CI 42%‐87%) of the mutations fell into one of the *BRCA2* OCCR, while the remaining 31% (95% CI 13%‐58%) were located in non‐*BRCA2* OCCR regions (Figure 2B). This distribution pattern was also found to be significantly different from OC patients with “OC+BC families”, where 29% (8/28; 95% CI 15%‐47%) of mutation carriers was located in the *BRCA2* OCCR (*P* = 0.020; Fisher's test).

### 3.3 Disease onset and familial history in OC patients

**Figure 3:**
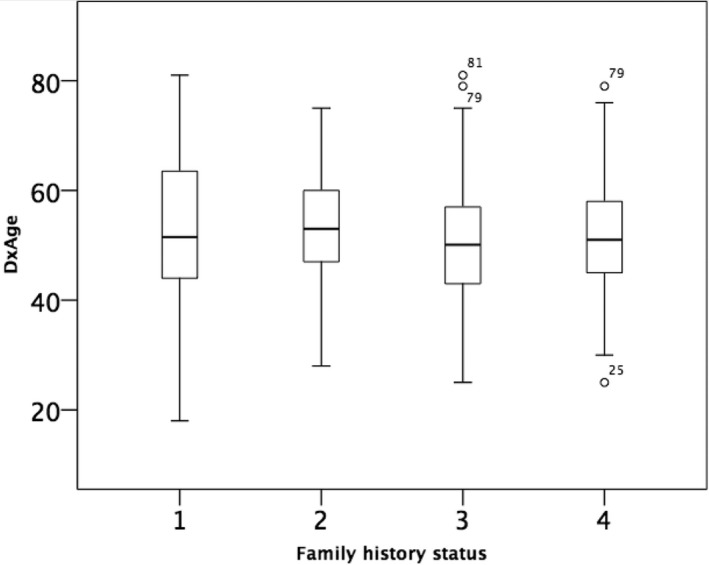
Age at diagnosis in OC patients with (1) no additional OC or BC in family; (2) at least one additional OC in family; (3) no additional OC but at least one BC in family; (4) at least one additional OC and at least one BC in family

**Figure 4:**
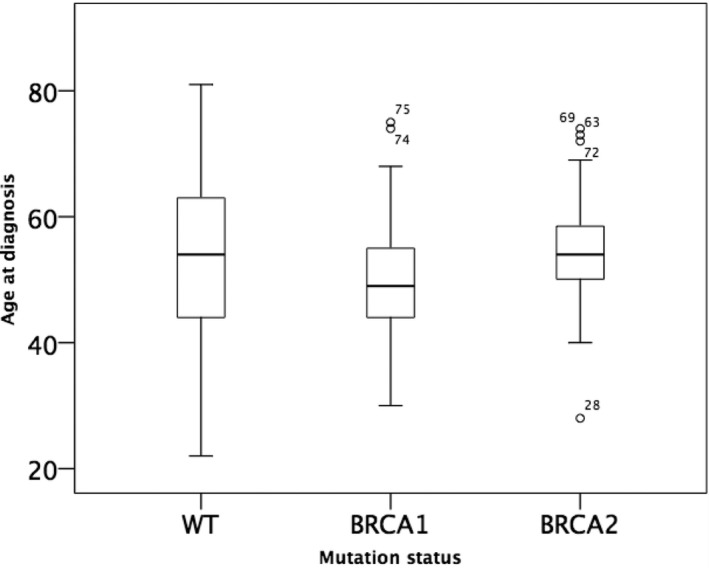
Age at diagnosis of OC patients with a *BRCA1*, *BRCA2* germline mutation, and in women without a *BRCA* germline mutation

We then looked at disease onset in women with *BRCA1* and *2 *germline mutations in relation to their family history. In women with sporadic OC, the mean age at diagnosis was 53 years (95% CI 51.1‐55.3 years) and was not significantly different from the mean age at diagnosis in women with at least one additional OC in the family, which was 53 years (95% CI 49.6‐56.4 years). Similarly, women who had developed OC but had no additional OC diagnosed in the family and OC patients with a familial history of both OC and BC were diagnosed at 52 (95% CI 49.5‐55.3 years) and 51 years (95% CI 49.6‐53.5 years), respectively (*P* = 0.341, one‐way ANOVA; Figure 3). When looking at disease onset stratified by g*BRCA* mutation status, we found that women with a *gBRCA1* mutation developed OC at a younger age (mean age at onset: 49 years (95% CI 48.2‐51.0 years), Figure 4) than women with a g*BRCA2 *mutation (mean age at onset: 54 years (95% CI 51.4‐57.8 years; *P* = 0.002, student's *t *test*)* as well as those without a germline mutation (mean age at onset: 53 years (95% CI 51.1‐55.3 years; *P* = 0.006, student's *t *test).

## 4 DISCUSSION

Until recently, relatively little is known about the predictive role of family history of cancer on the prevalence of germline *BRCA1* or *2* mutations in OC patients. Most of the information available is based on prevalence data in relation to tumor histology, while family history was either not available or based on arbitrary definitions in most publications.^15^, ^16^, ^17^, ^18^ Prevalence data therefore vary considerably: A population‐based study of 209 women with invasive OC treated in the Tampa Bay area found that 32 patients (15.3%) had mutations in *BRCA1* or *2*.^3^ A large kin‐cohort study conducted in Ontario in 977 patients with invasive OC observed a similarly high total mutation frequency for *BRCA1* and *2*, which was 13.2%.^19^ In contrast, a population‐based Danish study of 445 confirmed OC cases in which both DNA sequencing and MLPA analysis were conducted to analyze both *BRCA1* and *2* genes for coding sequence mutations and large genomic rearrangements detected deleterious mutation in only 5.8% OC cases.^20^ Contrary to our findings where the overall mutation rate was 42% and about 91% of all patients has either an OC or BC diagnosed in the family, Harter et al^21^ reported a rate of 21% (109/523) in patients with first diagnosis or relapsed disease, and only 43% of all patients have a positive family history, that is, at least 1 BC or OC in family or BC and OC in personal history. While Harter et al reported that patients with a positive family history had a 32% prevalence of deleterious variants, we found in our cohort of patients with a positive family history of cancer to carry a deleterious *BRCA1* or *2* to be as high as 51% (168/331; 95% CI 45%‐56%). The difference in mutation prevalence observed here may be due to different selection criteria used in both studies. The prevalence of mutations is also thought to be highly dependent on ethnicity and familial burden. Recently, Choi et al investigated the presence of germline *BRCA1* or *2* mutations in a small set of 70 Korean OC patients and their family history. Eighteen women had a family history of cancer, defined by either the presence of OC and BC in the tested individual or by a history of BC and/or OC in a first‐degree family member. With striking similarity to our findings, they reported that 11 of 18 (61.1%) with a family history of cancer carried *BRCA1* or *2* mutations, while only 7 of 52 (13.5%) without any family history did.^22^, ^23^ A family‐based assessment of *BRCA1* or *2* mutations conducted in 283 OC families in the United Kingdom and in the United States identified coding sequence changes and genomic rearrangements in 46% of the families. The prevalence of germline *BRCA1* or *2* mutations correlated with the extent of ovarian and breast cancer in families and was particularly high (81%) in families containing more than two OC cases and at least one case of BC diagnosed before age 60.^24^ Extremely high mutation frequencies were also reported in a historical collection of families particularly burdened by breast and ovarian cancer. In this set of families with an average of 2.8 OC cases and 4.9 BC cases per family, *BRCA1* or *2* mutations were detected in 84% of families.^25^ In both analyses, however, and in contrast to our study, the familial burden rather than the probability of individual OC patients was assessed. In another large study of 21,401 families with BC and OC in Germany, the authors identified an overall mutation prevalence of 24%, with highest mutation frequencies observed in families with at least two OCs (41.9%) and families with at least one BC and one OC (41.6%).^23^

Our finding of an earlier onset of OC in women with a *BRCA1* mutation is consistent with other studies in this field. Riesch et al, for example, have tested a panel of 11 of the most commonly reported *BRCA1* or *2 *mutations in a population‐based series of 515 incident cases of invasive ovarian cancer diagnosed in Ontario. The average age at diagnosis of the 39 *BRCA1*‐positive cases was 51.2 years, which was significantly younger than the average age at diagnosis in the 21 *BRCA2* carriers who developed OC at 57.5 years, and in the average age at diagnosis of the 455 women without BRCA mutation was 55.6 years.^26^ In another study, which was restricted to an analysis of six of the most common *BRCA1* mutations in 698 consecutive OC patients, the mean age of onset in mutation carriers with a familial background was 48.5 years. *BRCA1 *mutation carriers without affected family members developed OC at a mean age of 54.2 years. Women who did not carry any of the investigated mutations had a mean age of 49 in the presence of a family history and a median age of 59.1 in the absence of a familial history.^27^

Our findings have important implications for genetic counseling of OC patients and their family members; because of the availability of PARP inhibitors, genetic counseling and the offer for genetic testing are now important for determining optimal therapy in all women with epithelial OC. This fact is increasingly reflected by a number of national and international guidelines.^2^, ^9^, ^10^, ^11^ In the face of increasing healthcare costs, it is important to determine whether relatives of women who have developed OC should also undergo genetic testing.^28^ This is particularly important for daughters of deceased OC patients who want to know their mutation status. Since, in most countries, the threshold for testing is set at a 10% pretest probability,^29^ our results would argue that testing OC patients based on family history criteria and/or age at onset is insufficient to identify all *BRCA1* or *2* carriers. In our cohort, 16 of 184 patients (9%; 95% CI 5%‐14%) with deleterious *BRCA1* or *2* mutation would have been missed if they were tested based on the presence of family history of cancer. Thus, testing should be offered to all OC patients until a better alternative for identifying families at high risk of ovarian cancer is found. We currently do not have evidence that healthy children from OC patients without any family history of cancer should be tested. However, if at least another family member has been diagnosed with breast or ovarian cancer, the pretest probability rises significantly, and testing should be considered.

Our study has several limitations; we have no information on whether the reported OC was of high‐ or low‐grade histology. Therefore, we are not able to provide a estimate prevalence for each subtype. Also, despite our prospective study design, we do not know whether families with nonhereditary OC have other family members will develop BC or OC later in life and would therefore be reclassified into another risk group. However, to ensure that all new cancer incidences within the families are captured, these families are followed over time with surveys every 2 years. Further follow‐up of the patients is planned and will be reported later.

In conclusion, we have demonstrated the role of family history of cancer in women diagnosed with OC for assessing the likelihood of a germline *BRCA1* or *2* mutations status. There will be a subgroup of *BRCA1* or *2*‐positive patients with ovarian cancer without a family history of cancer, and these patients would have been missed if using family history of cancer and/or age at onset as selection criteria for testing. Thus, this highlights the necessity to offer germline testing to all patients with ovarian cancer. To our knowledge, this is one of the largest studies to date in OC patients in which both, prospectively collected BC and OC family history data and complete *BRCA1 *and *2* sequencing and MLPA data, are available. Our results will direct genetic counseling and/or genetic testing for family members of OC patients whose *BRCA1* and *2* testing results are unavailable either because they have died or refused genetic testing.

## CONFLICTS OF INTEREST

None.
